# Supplementary material for: A Review of the COVID-19 Mental Health Impact in Post-Conflict Settings: Bridging the Mental Health Gap with Case Exemplars from an Implementation Science Lens
Source: Int J Environ Res Public Health. 2023 May 31;20(11):6006. doi: 10.3390/ijerph20116006 (PMC10252255; doi:10.3390/ijerph20116006)
Supplement: Supplementary file 1 [file ijerph-20-06006-s001.zip › ijerph-2299756-supplementary.pdf]

Supplementary Materials

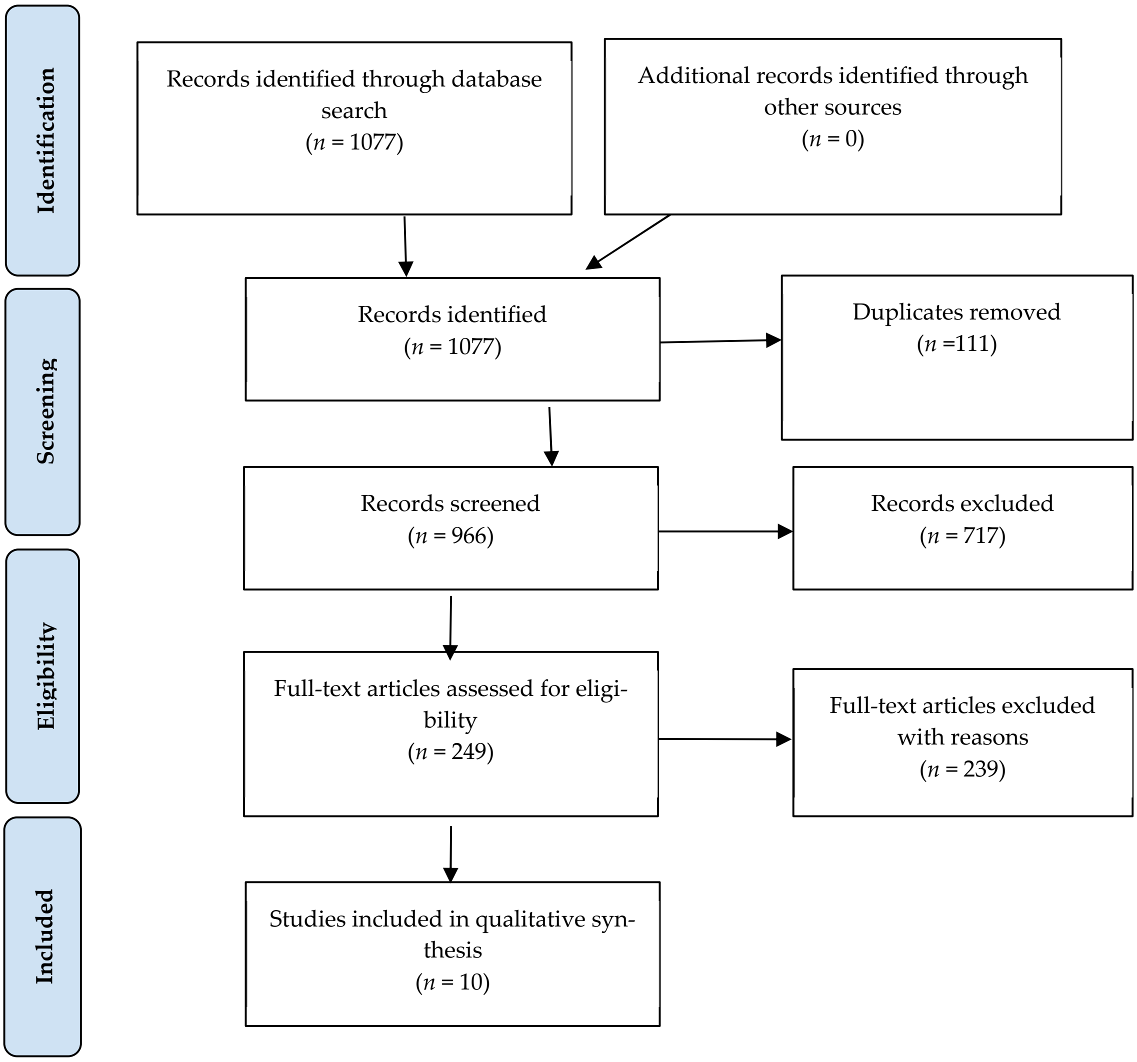

Figure S1. PRISMA Flow Diagram.

Table S1. Review Methodology.

Databases	PubMed, Cochrane Library, Embase, Web of Science, and APA PsycInfo
Search Terms	"Mental health" OR "anxiety" OR "depression" OR "PTSD" OR "stress" OR "mental disorder*" OR "trauma") AND ("COVID") AND ("inequity" OR "resource constrained" OR "low resource" OR "poverty" OR "resource-poor" OR "LMIC*" OR "low-income country*" OR "developing" OR "underserved" OR "marginalized" OR "post-conflict") AND ("psychological" OR "psychiatric" OR "wellbeing")
Inclusion Criteria	• Adults 18+<br>• Published in English<br>• 2019+<br>• Resource-poor setting, post-conflict settings<br>• Mental health disorders<br>• Novel evidence-based interventions<br>• COVID
Exclusion Criteria	• Children<br>• Not published in English<br>• World bank defined HIC, settings without conflict<br>• Mental illness<br>• Not evidence-based

Table S2. Analysis of Studies Included in Qualitative Synthesis.

Author	EBI Exemplar	Country	Objective	Study Design	Method	Outcomes/Recommendations
Abas, et al. (2016)	Yes	Zimbabwe	Improve primary mental health service access through collaborative care	Pilot study	Focus group discussion identified level of intervention acceptability and implementation effectiveness	The Friendship Bench uses trained community lay workers to deliver mental health screening and problem-solving psychotherapy. The study showed this to be an effective intervention by increased patient acceptability, low cost, and sustainability for local service delivery infrastructure
Alonzo, et al. (2021)	Yes	Guatemala	Improve recognition of mental health symptoms, promote help seeking, and provide immediate strategies for self-care for individuals experiencing psychological distress related to the COVID-19 pandemic in low-income, high-risk communities in and around Guatemala city.	Participatory approach	5×5 social media campaign across several different social media groups such as WhatsApp, Instagram, and Facebook	Social media should be utilized more frequently to further develop awareness of mental health and to break the stigma in seeking help for underserved populations. Mental health struggles amongst resource-limited communities are severely underserved and should be viewed as a complex emergency to foster further study upon the issue. Poor emotional health leads to higher vulnerabilities, which ultimately impacts an individual's socioeconomic conditions.
Arenliu, et al. (2020)	Yes	Kosovo	Summarize the experiences learned in using internet and phone services in provision of mental health services during pandemics in LMIC settings and strives	Case Report/Study	Development and lessons learned in deployment of online and telephone based psychological first aid	The study showed that online and telephone based psychological first aid is an effective method of improving mental health service delivery in disastrous circumstances

			to recommend how lessons learned can serve for future emergencies.			
Jemal et al. (2021)	No	Ethiopia	Assess the prevalence of anxiety and depression symptoms in older adults during the COVID-19 pandemic in Ethiopia	Cross-sectional study	Elderly at home interviews consisted of standardized measuring tools such as the Geriatric Depression Scale and the General Anxiety Disorder-7 Scale	The study showed older adults had higher rates of depression and anxiety during and after the COVID-19 pandemic. Associated predisposing factors were identified as chronic comorbidities and lacking social support. There must be more targeted mental health interventions for the elderly that ensure this population is not neglected in future disasters
Kola, et al. (2021)	No	Low-income and middle-income countries	Examine the global mental health implications of the COVID-19 pandemic in four parts: first, the impact of the pandemic on mental health; second, the responses in different countries; third, the opportunity that the pandemic presents to reimagine global mental health; and finally, a future vision for mental health systems	Review	Analysis of mental health-related literature in LMICs, specifically identifying short- and long-term mental health impacts due to COVID-19, the different approaches that were used, and placing an emphasis on the importance of global mental health.	Build Back Better: Integrating mental health services into UHC, Improving access and coverage of psychosocial interventions, Eliminating coercion in mental health care, Integrating mental health interventions into other sectors, Addressing the mental health of children and young people, Incorporating technological innovations in mental health services.

Kwobah, et al. (2021)	No	Kenya	Describe the efforts of ensuring mental healthcare delivery is continued in a referral hospital in Kenya as well as the challenges faced.	Case Report/Study	Kept the hospital open but reduced maximum capacity, limited the mental health unit to only those severely agitated, trained staff on COVID-19 control and prevention measures, temporary primary care workers in peripheral hospitals to manage mentally ill patients, allocated a mental health nurse to follow up with mentally ill patients who missed appointments, and promoted mental wellness among staff and general public.	Several factors contribute to disruption in ensuring continuity of mental healthcare in a low resource setting: Limited ability to move about, Declining social economic status, Stigma associated with mental illness leading to poor social support, Limited drug supply in peripheral facilities, Low internet availability for tele-health, Difficulties of mentally ill patients to adhere with infection control measures, Staff Shortages, and Limited access to addiction services.<br><br>The government should provide stimulus packages to help institutions meet the rising cost of care, greater involvement of community-based healthcare, more trainings provided to healthcare workers in peripheral hospitals to limit the need for referrals, explore ways to strengthen community pharmacists, and partner with other stakeholders (NGOs, private facilities) to increase access.
Leochico, et al (2020)		Philippines	Determine the challenges faced by telerehabilitation in the Philippines.	Systematic Review	Analysis of telerehabilitation-related publications (including gray literature) wherein Filipinos were involved as investigator or population.	The most common challenges identified in the review were slow internet speed (in 10 studies), legal concerns (9), and skepticism (9).

Lin, et al. (2022)	No	Iran	Examine the associations between generalized trust, fear of COVID-19, insomnia severity, and suicidal ideation.	Cluster-sampling	Standardized survey tools were used such as the Patient Health Questionnaire (PHQ-9) to measure suicidal ideation, Insomnia Severity Index, Generalized Trust Scale, and Fear of COVID-19 Scale	The current study suggests that generalized trust might reduce levels of fear of COVID-19 and insomnia and consequently might decrease suicidal ideation. Recommends healthcare providers may want to find ways to improve generalized trust to resolve the mental health problems during the COVID-19 pandemic.
Nkodila, et al. (2021)	No	Democratic Republic of Congo	Assess the psychological aspects of the COVID-19 pandemic in the population of Kinshasa.	Analytical cross-sectional study	Population survey from Oct - Nov 2020	More attention should be paid to vulnerable groups (old people, women, and people with a comorbidity history).
Nwafor, et al. (2021)	No	Nigeria	Determine the prevalence and predictors of COVID-19-related depression, anxiety and stress symptoms among pregnant women	Cross-sectional study	Patients were screened for psychological morbidities using the Depression Anxiety and Stress Scale-21 (DASS-21).	Need to integrate screening for depression, anxiety and stress, in existing antenatal care programs so as to identify and prevent long-term adverse psychological outcomes related to the COVID-19 pandemic.

EBI Exemplar Selection Criteria:

1) Case study interventions that possess strong innovative advantages for different populations and care levels

2) Interventions adaptable to disastrous events ensuring continued service delivery

3) Describe implementation outcomes applicable to similar settings
